# Right bundle branch block is not associated with worse short- and mid-term outcome after transcatheter aortic valve implantation

**DOI:** 10.1371/journal.pone.0253332

**Published:** 2021-06-16

**Authors:** Maren Weferling, Christoph Liebetrau, Matthias Renker, Ulrich Fischer-Rasokat, Yeoung-Hoon Choi, Christian W. Hamm, Won-Keun Kim

**Affiliations:** 1 Kerckhoff Heart and Thorax Center, Department of Cardiology, Bad Nauheim, Germany; 2 German Centre for Cardiovascular Research (DZHK), Partner Site RheinMain, Berlin, Germany; 3 Cardioangiological Center Bethanien (CCB), Department of Cardiology, Agaplesion Bethanien Hospital, Frankfurt, Germany; 4 Kerckhoff Heart and Thorax Center, Department of Cardiac Surgery, Bad Nauheim, Germany; 5 Department of Cardiology, University Hospital of Giessen, Giessen, Germany; Klinikum Region Hannover GmbH, GERMANY

## Abstract

**Background:**

Transcatheter aortic valve implantation (TAVI) is the standard treatment option for patients with severe aortic stenosis (AS) at intermediate or high surgical risk. Preexisting right bundle branch block (RBBB) is a strong predictor of new pacemaker implantation (PPM) after TAVI, and previous data indicate a worse short- and long-term outcome of patients. The aim of this study was to investigate whether preexisting RBBB has an effect on the short- and mid-term outcome of patients undergoing TAVI in a German high-volume TAVI center.

**Methods:**

For the present retrospective analysis, a total of 1,891 patients with native severe AS with successful TAVI without preexisting PPM were included. The primary endpoint was all-cause mortality after 30 days and 12 months. Baseline RBBB was present in 190 (10.1%) of cases.

**Results:**

Patients with preexisting RBBB had a considerably higher rate of new PPM after TAVI compared with patients without RBBB (87/190 [45.8%] vs. 219/1,701 [12.9%]; p<0.001). RBBB had no impact on all-cause mortality at 30 days (2.1% vs. 2.7%; p = 0.625) and at 12 months (14.4% vs. 13.6%; p = 0.765). Further stratification according to the presence of new PPM showed a difference in mid-term survival rates between the four groups, with the worst outcome for patients without RBBB and new PPM (log rank p = 0.024). However, no difference in mid-term cardiovascular survival was found.

**Conclusion:**

Preexisting RBBB is a common finding in patients with severe AS undergoing TAVI and is associated with considerably higher PPM rates but not with worse short- and mid-term outcome.

## Introduction

During the last two decades transcatheter aortic valve implantation (TAVI) has evolved as a standard treatment option in patients with symptomatic severe aortic stenosis (AS) who are at increased surgical risk [1–4]. While the need for implantation of a permanent pacemaker (PPM) due to conduction disturbances is described in 5–8.9% of cases after surgical valve replacement [1–3], PPM rates range between 2% and 51% after TAVI, depending on the prosthesis type and procedural factors [5].

Currently, up to 19,000 TAVI procedures are performed in Germany annually [6]. Preexisting right bundle branch block (RBBB) before TAVI has been reported to have a prevalence between 10.3% and 13.6% [7–9] and was found to be a dominant predictor of new PPM implantation after TAVI [8, 10]. In the literature, only two studies to date have examined the outcome of patients with preexisting RBBB undergoing TAVI; these uniformly indicate a worse short- and long-term outcome in this subgroup compared with patients without RBBB [7, 9]. The reasons for the higher all-cause and cardiovascular mortality rates have not yet been fully identified, and there is an ongoing debate regarding the impact of PPM on outcome in this subgroup. The aim of the present study was to examine the prevalence of preexisting RBBB in TAVI patients, PPM rates after TAVI, and the association of RBBB with short- and mid-term outcome at a German high-volume TAVI center.

## Methods

Between January 2010 and April 2019, a total of 2,346 patients with symptomatic severe native AS planned for transfemoral TAVI were enrolled in a German single-center TAVI-registry. Patients were found to be eligible for TAVI based on the clinical consensus within a multidisciplinary heart team consisting of interventional cardiologists, cardiothoracic surgeons, and anesthesiologists. Exclusion criteria for this retrospective analysis were preexisting PPM (n = 393) and periprocedural conversion to surgical aortic valve replacement (n = 62). Hence, the final cohort comprised 1,891 patients for the analysis. Balloon-expandable (Edwards Sapien XT, Edwards Sapien 3, and Edwards Sapien 3 Ultra; Edwards Lifesciences, Irvine, California, USA) and self-expanding ((Symetis neo, Symetis ACURATE, ACURATE Neo (Boston Scientific, Marlborough, MA, USA), CoreValve, CoreValve Evolut (Medtronic Inc., Dublin, Ireland), Portico (Abbott, Chicago, Illinois, USA)) and mechanically expanding (Lotus; Boston Scientific, Marlborough, MA, USA) THV types were implanted. For practical purposes, the Lotus THV (accounting for only 1% of implanted valves in the present analysis) was assigned to the group of self-expanding valves. A baseline 12-lead electrocardiogram was obtained, and conduction disturbances were diagnosed according to current guideline recommendations [11]. Procedural outcomes and complications were defined according to the Valve Academic Research Consortium (VARC) II criteria [12]. The decision regarding PPM implantation was made on the basis of the current guideline recommendations [13]. Follow-up data were obtained via outpatient visits, telephone interview, or from medical reports from referring hospitals/general practitioners. The primary endpoint of the study was all-cause mortality after 30 days and one year and the secondary endpoint was cardiovascular mortality after one year. The study was conducted in adherence to the Declaration of Helsinki and was approved by the Ethics Committee of the University of Giessen, Germany (AZ 180/20).

Continuous variables are presented as mean with standard deviation (SD) or as median with interquartile range (IQR), as appropriate. Categorical variables are given as frequencies and percentages. The presence of a normal distribution pattern was tested using the Kolmogorov-Smirnov test. The Mann-Whitney-U test was used for comparison of continuous variables. For comparison of categorical variables, the chi-squared test was applied.

Univariate Cox regression was used to identify independent predictors of all-cause mortality. Covariates with a p-value of 0.05 or lower were further analyzed in multivariate Cox regression. The following variables were included for Cox regression analysis: age, sex, body mass index (BMI, kg/m^2^), New York Heart Association (NYHA) functional class >II, logistic EuroScore I and EuroScore II, syncope, prior cardiac decompensation, baseline ejection fraction and mean transvalvular gradient, diabetes, chronic obstructive pulmonary disease, atrial fibrillation, coronary artery disease, prior myocardial infarction, peripheral artery disease, history of coronary artery bypass graft, preexisting left bundle branch block (LBBB) and RBBB, preexisting atrioventricular block (AVB) I°, periprocedural major/life-threatening bleeding and major vascular complications, disabling stroke after TAVI, acute kidney injury (AKI) stage ≥2, paravalvular leakage (PVL) ≥moderate, and new PPM implantation. The proportional hazard assumption was present in every variable tested except for age, major/life-threatening bleeding, major vascular complications, disabling stroke after TAVI, PVL ≥moderate, and AKI >stage 2.

Estimated survival rates on the basis of the presence of RBBB were determined by the Kaplan-Meier method with consecutive comparison by utilization of log-rank-test.

Significance was assumed when a two-sided p-value <0.05 was determined. SPSS Version 22.0 (IBM, Armonk, New York, USA) was used for all statistical analyses.

## Results

### Patient characteristics

A total of 1,891 patients (56.3% female) with a median age of 82 years [IQR 79–85] were included. Table 1 shows the patient characteristics of the entire cohort and the non-RBBB and RBBB subcohorts. In 190 patients (10.1%), RBBB was present before TAVI. There was a male predominance in the RBBB group compared with the non-RBBB group (66.3% vs. 41.2%; p<0.001). The median EuroScore II was lower in the RBBB cohort (3.3% [IQR 2.3–6.2] vs. 4.4% [IQR 2.6–7.3]; p = 0.002). Coronary artery disease and previous percutaneous coronary intervention were more frequent in the RBBB group than in the non-RBBB group (66.3% vs. 57.4%; p = 0.018; 41.6% vs. 33.5%; p = 0.026). Fewer patients in the RBBB group had atrial fibrillation and arterial hypertension than in the non-RBBB group (30.0% vs. 38.7%; p = 0.019; 87.9% vs. 92.3%; p = 0.035), and first-degree AV block was more common in RBBB patients (28.4% vs. 16.8%; p<0.001).

**Table 1:** Baseline characteristics.

	Total cohort	non-RBBB	RBBB	p-value
	(n = 1,891)	(n = 1,701)	(n = 190)	
Age, years	82 [79–85]	82 [79–85]	83 [79–86]	0.064
Sex, male	827 (43.7)	701 (41.2)	126 (66.3)	*<0*.*001*
Body mass index, kg/m^2^	26.8 [24.1–30.4]	26.8 [24.0–30.5]	26.9 [24.3–29.9]	0.885
NYHA class 3 or 4	1521 (80.4)	1377 (81.0)	144 (75.8)	0.089
Syncope	287 (15.2)	268 (15.8)	19 (10.0)	*0*.*036*
Cardiac decompensation	537 (28.4)	489 (28.7)	48 (25.3)	0.312
Hypertension	1737 (91.9)	1570 (92.3)	167 (87.9)	*0*.*035*
Diabetes mellitus	604 (31.9)	545 (32.0)	59 (31.1)	0.782
Hyperlipidemia	704 (37.2)	623 (36.6)	81 (42.6)	0.104
Logistic EuroScore I, %	18.54 [12.2–27.1]	18.6 [12.2–27.1]	18.2 [12.9–26.6]	0.878
EuroScore II, %	4.4 [2.6–7.2]	4.4 [2.6–7.3]	3.3 [2.3–6.2]	*0*.*002*
Coronary artery disease	1102 (58.3)	976 (57.4)	126 (66.3)	*0*.*018*
Previous MI	212 (11.2)	194 (11.4)	18 (9.5)	0.424
Previous PCI	649 (34.3)	570 (33.5)	79 (41.6)	*0*.*026*
Peripheral artery disease	213 (11.3)	194 (11.4)	19 (10.0)	0.561
eGFR, ml/min/1.73m^2^	67 [48–87]	67 [48–86]	72 [51–91]	0.109
COPD	376 (19.9)	330 (19.4)	46 (24.2)	0.115
Prior stroke	234 (12.4)	209 (12.3)	25 (13.2)	0.729
Atrial fibrillation	715 (37.8)	658 (38.7)	57 (30.0)	*0*.*019*
AV block I°	340 (18.0)	286 (16.8)	54 (28.4)	*<0*.*001*
LBBB	190 (10.0)	190 (10.0)	--	--
CABG	209 (11.1)	187 (11.0)	22 (11.6)	0.807
Ejection fraction, %	65 [55–65]	65 [55–65]	65 [50–65]	0.508
Pmean, mmHg	43 [34–54]	43 [34–54]	42 [35–53]	0.474
Aortic valve area, cm^2^	0.7 [0.5–0.8]	0.7 [0.5–0.8]	0.7 [0.6–0.8]	*0*.*047*

Values denote number (%) or median [interquartile range].

Abbreviations: NYHA class = New York Heart Association class, MI = myocardial infarction, PCI = percutaneous coronary intervention; eGFR = estimated glomerular filtration rate; COPD = chronic obstructive pulmonary disease; AV block I° = atrioventricular block I°; LBBB = left bundle branch block; CABG = coronary artery bypass graft; Pmean = mean transvalvular aortic pressure

### Procedural and postprocedural characteristics

In the total cohort, self-expandable valves were more frequently used than balloon-expandable valves (63.1% and 36.9%); however, in the RBBB group, more balloon-expandable valves were implanted (43.7% vs. 36.1%; p = 0.04). S1 Table in the *supporting information* depicts the distribution pattern of the different transcatheter heart valves in the total cohort and the non-RBBB and RBBB groups.

Table 2 presents the procedural and postprocedural characteristics of the total cohort and the RBBB and non-RBBB groups. There were no differences in the incidence of major or life-threatening bleeding or in major vascular complications between the groups. Rates for device embolization, left ventricular injury, and aortic root injury were similar.

**Table 2:** Procedural and postprocedural characteristics and outcomes.

	Total cohort	non-RBBB	RBBB	p-value
	(n = 1,891)	(n = 1,701)	(n = 190)	
***Length of hospital stay*, *days***	8 [7–12]	8 [7–11]	9 [7–12]	*0*.*019*
***Prothesis type***				*0*.*040*
Balloon-expandable	697 (36.9)	614 (36.1)	83 (43.7)	
Self-expanding	1194 (63.1)	1087 (63.9)	107 (56.3)	
Implantation depth, mm	5 [3–6]	5 [3–6]	5 [3–6]	0.975
***Procedural and post-procedural complications***				
Major/life-threatening bleeding	116 (6.1)	107 (6.3)	9 (4.7)	0.397
Major vascular complications	152 (8.0)	142 (8.3)	10 (5.3)	0.138
Valve embolization	25 (1.3)	23 (1.4)	2 (1.1)	0.732
Left ventricular injury	1 (0.1)	1 (0.1)	0 (0)	0.738
Aortic root injury	8 (0.4)	7 (0.4)	1 (0.5)	0.817
Valve-in-valve	41 (2.2)	37 (2.2)	4 (2.1)	0.950
Device success	1675 (88.6)	1500 (88.2)	175 (92.1)	0.107
Disabling stroke	53 (2.8)	48 (2.8)	5 (2.6)	0.885
AKI stage 2 or 3	52 (2.8)	44 (2.6)	8 (4.2)	0.195
New PPM	306 (16.2)	219 (12.9)	87 (45.8)	*<0*.*001*
***Postprocedural echocardiographic findings***				
Ejection fraction, %	65 [60–65]	65 [60–65]	65 [55–65]	0.438
Pmean, mmHg	10 [7–13]	10 [7–13]	9 [7–13]	0.996
Aortic valve area, cm^2^	1.6 [1.4–1.8]	1.6 [1.4–1.8]	1.7 [1.5–1.9]	*0*.*007*
Paravalvular leakage ≥moderate	63 (3.4)	59 (3.5)	4 (2.1)	0.322
***Follow-up***				
30-day all-cause mortality	50 (2.6)	46 (2.7)	4 (2.1)	0.625
1-year all-cause mortality	252 (13.7)	225 (13.6)	27 (14.4)	0.765
1-year cardiovascular mortality	166 (9)	149 (9)	17 (9)	0.901

Values denote number (%) or median [interquartile range].

Abbreviations: RBBB = right bundle branch block; AKI = acute kidney injury; Pmean = mean pressure gradient; PPM = permanent pacemaker

A total of 306 patients (16.2%) were implanted with a new PPM after TAVI, and there was a more than threefold higher rate in the RBBB group than in the non-RBBB group (45.8% vs. 12.9%; p <0.001) (Table 2). The median time from procedure to PPM implantation was 3 days [IQR 1–7] for the entire cohort. The median time from procedure to PPM implantation was significantly shorter in the RBBB group (1 day [IQR 1–4] vs. 4 days [IQR 2–7]; p<0.001).

### Outcome data

The clinical follow-up rate was complete (100%) at 30 days; 45 patients (2.4%) were lost to follow-up at one year after TAVI. After 30 days, 50 patients (2.6%) of the overall cohort had died: 2.7% (46/1,701) in the non-RBBB group and 2.1% (4/190) in the RBBB-group (p = 0.625) (Table 2). There was no difference in short-term outcome between RBBB patients with or without new PPM compared with non-RBBB patients with or without new PPM (death rates for RBBB-/PPM-: 2.6%; RBBB-/PPM+: 3.7%; RBBB+/PPM-: 1.9%; RBBB+/PPM+: 2.3%; log-rank p = 0.776).

**Fig 1:**
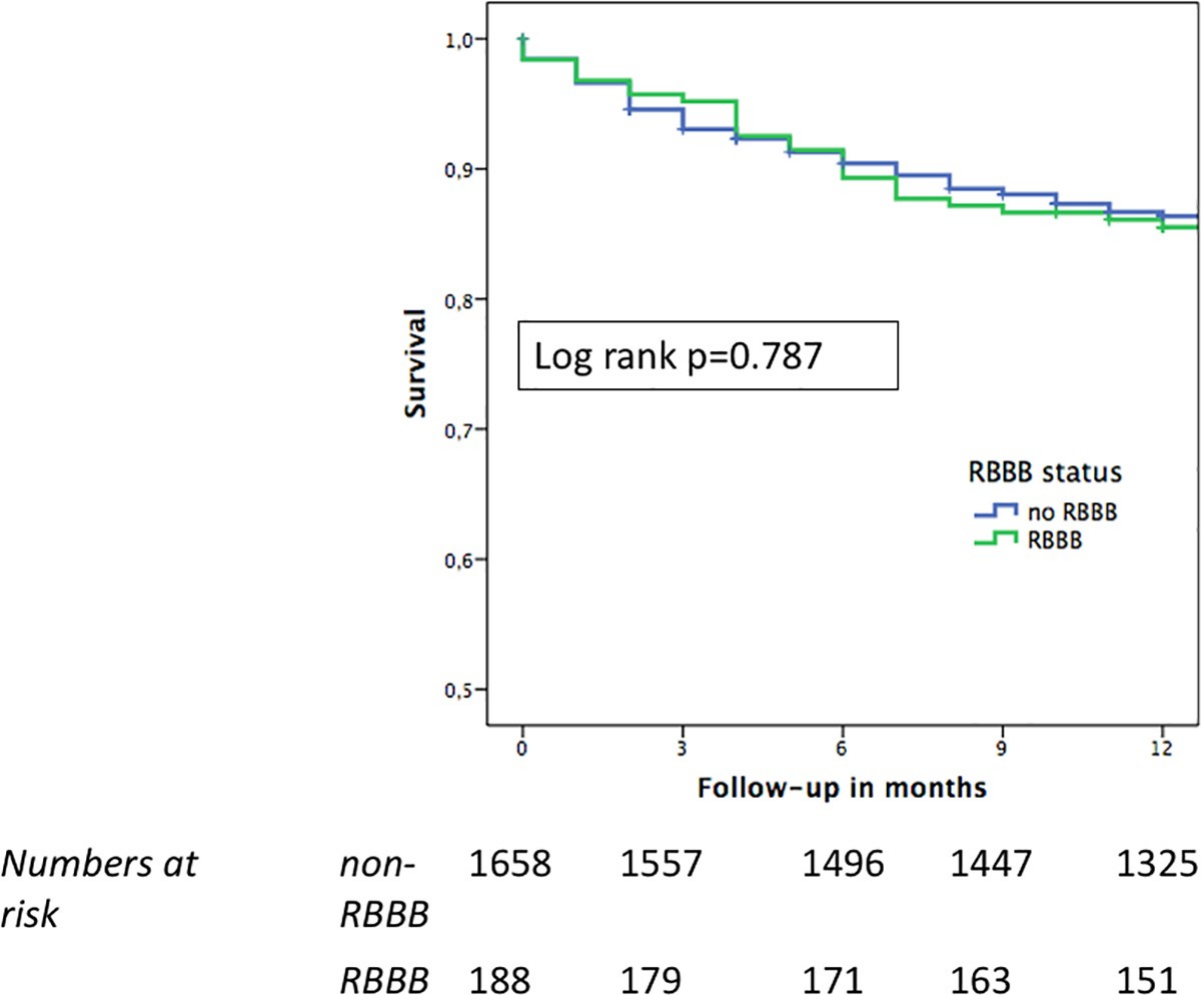
Kaplan-Meier estimates for one-year survival in patients with baseline RBBB versus no RBBB. During a follow-up time of 12 months, no difference in survival was observed between RBBB and non-RBBB patients. Abbreviations: RBBB = right bundle branch block.

**Fig 2:**
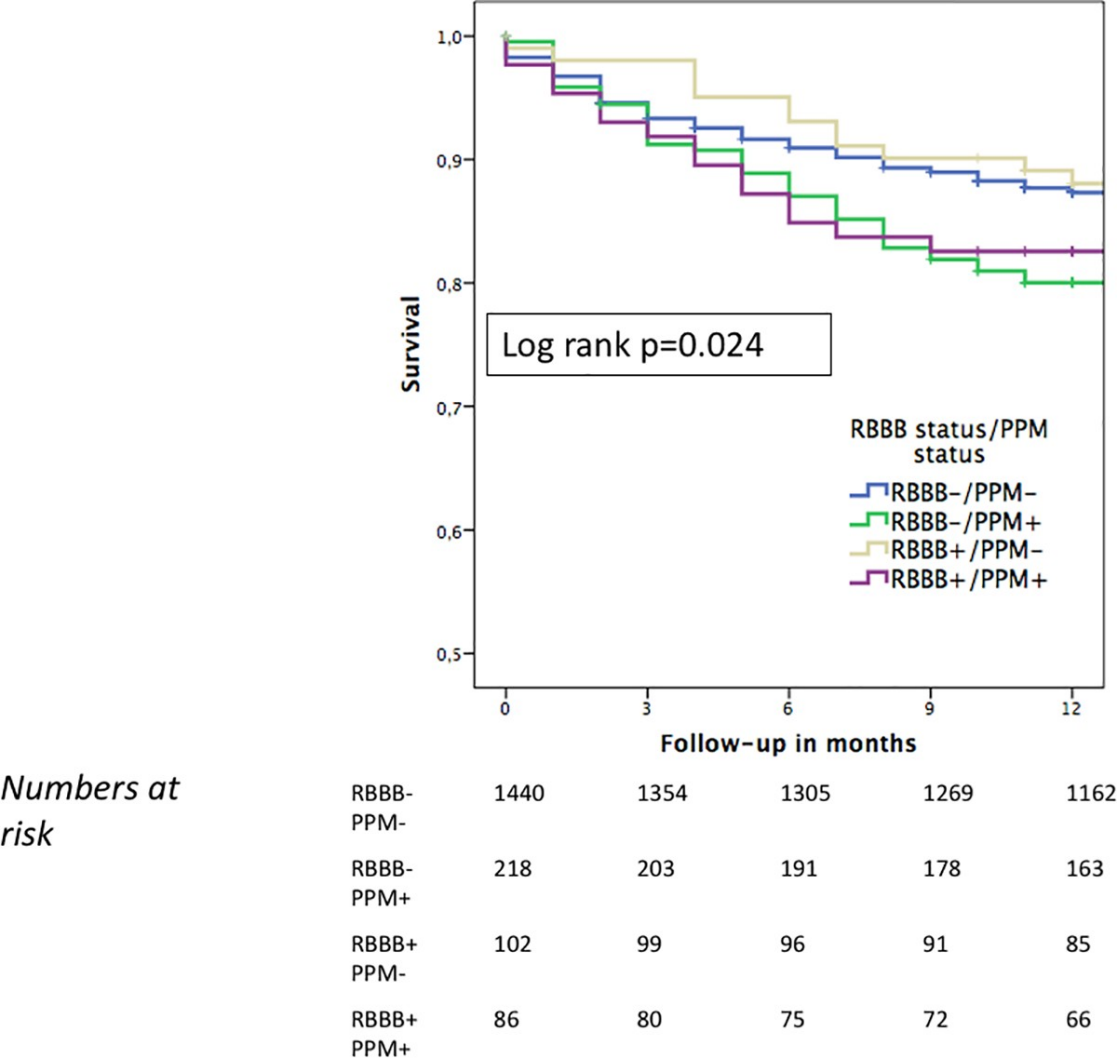
Kaplan-Meier estimates for one-year survival in RBBB versus non-RBBB patients stratified according to PPM implantation. During a follow-up time of 12 months, there was a significant difference in survival probability between the subgroups of RBBB and non-RBBB patients with either a new PPM or no new PPM: the worst outcome was observed for non-RBBB patients with new PPM. Abbreviations: RBBB = right bundle branch block; PPM = permanent pacemaker.

Within the follow-up time of 12 months, 252/1846 patients (13.7%) in the entire cohort had died: 225/1658 patients in the non-RBBB group and 27/188 patients in the RBBB group (13.6% vs. 14.4%; p = 0.765) (Table 2). The Kaplan-Meier survival curves revealed no difference in overall survival rates between the two groups during the follow-up time of 12 months (log-rank p = 0.787) (Fig 1). However, further stratification of the Kaplan-Meier survival analysis according to PPM revealed a significant difference among the 4 subgroups (log rank p = 0.024) (Fig 2), with non-RBBB patients with new PPM having the worst survival of all: death rates were 12.6% and 19.7% for the non-RBBB cohort without and with new PPM, respectively, and 11.8% and 17.4% for the RBBB group without and with new PPM, respectively. When considering the non-RBBB and RBBB cohorts separately and comparing patients with new PPM versus those without new PPM, in the non-RBBB group the overall survival in the subgroup without new PPM was significantly better (log rank p = 0.005) whereas in the RBBB group there was a numerically but not statistically significant difference in survival between patients with new PPM and those without new PPM (log rank p = 0.246) (S1 and S2 Figs).

**Fig 3:**
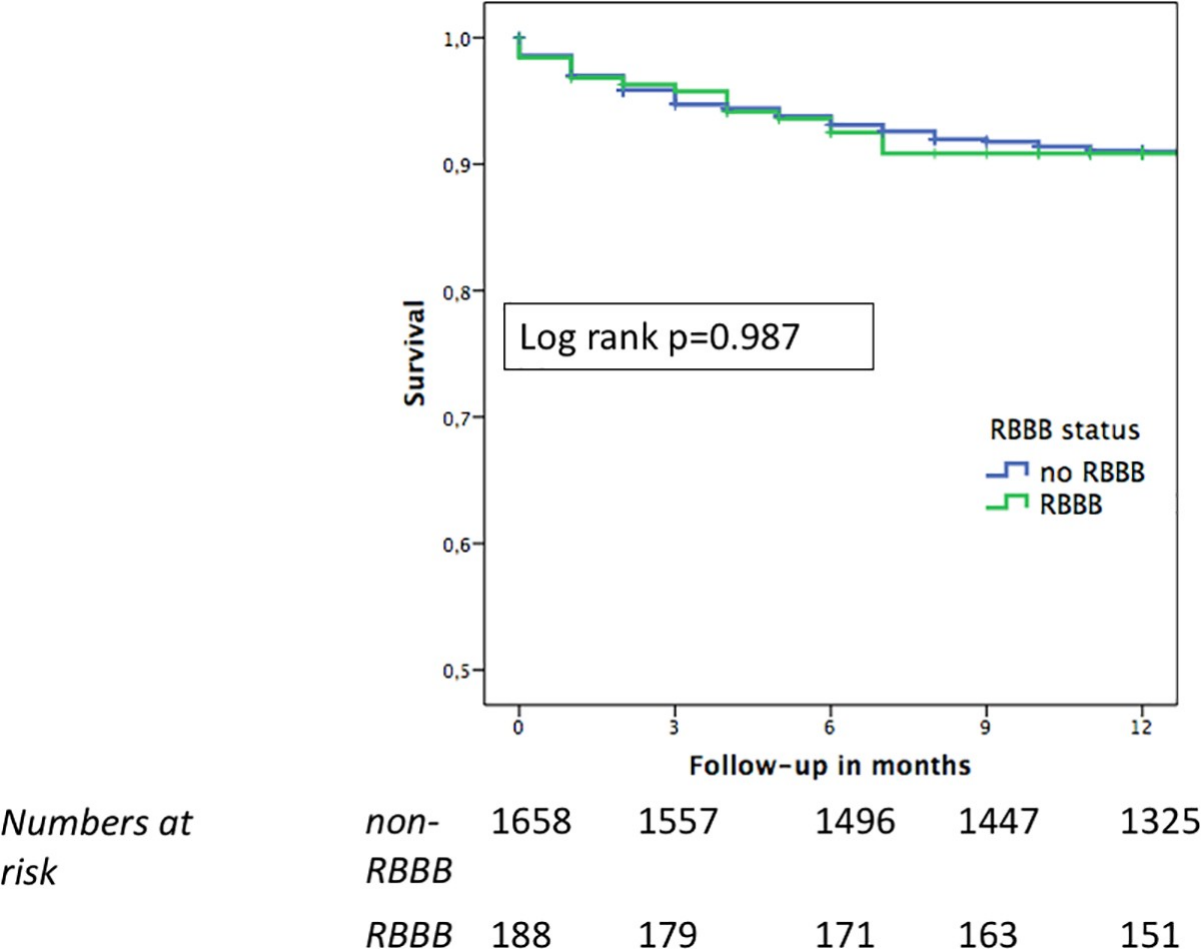
One-year cardiovascular survival in patients with baseline RBBB or no RBBB. During a follow-up time of 12 months, no difference in cardiovascular survival was observed between RBBB and non-RBBB patients. Abbreviations: RBBB = right bundle branch block.

**Fig 4:**
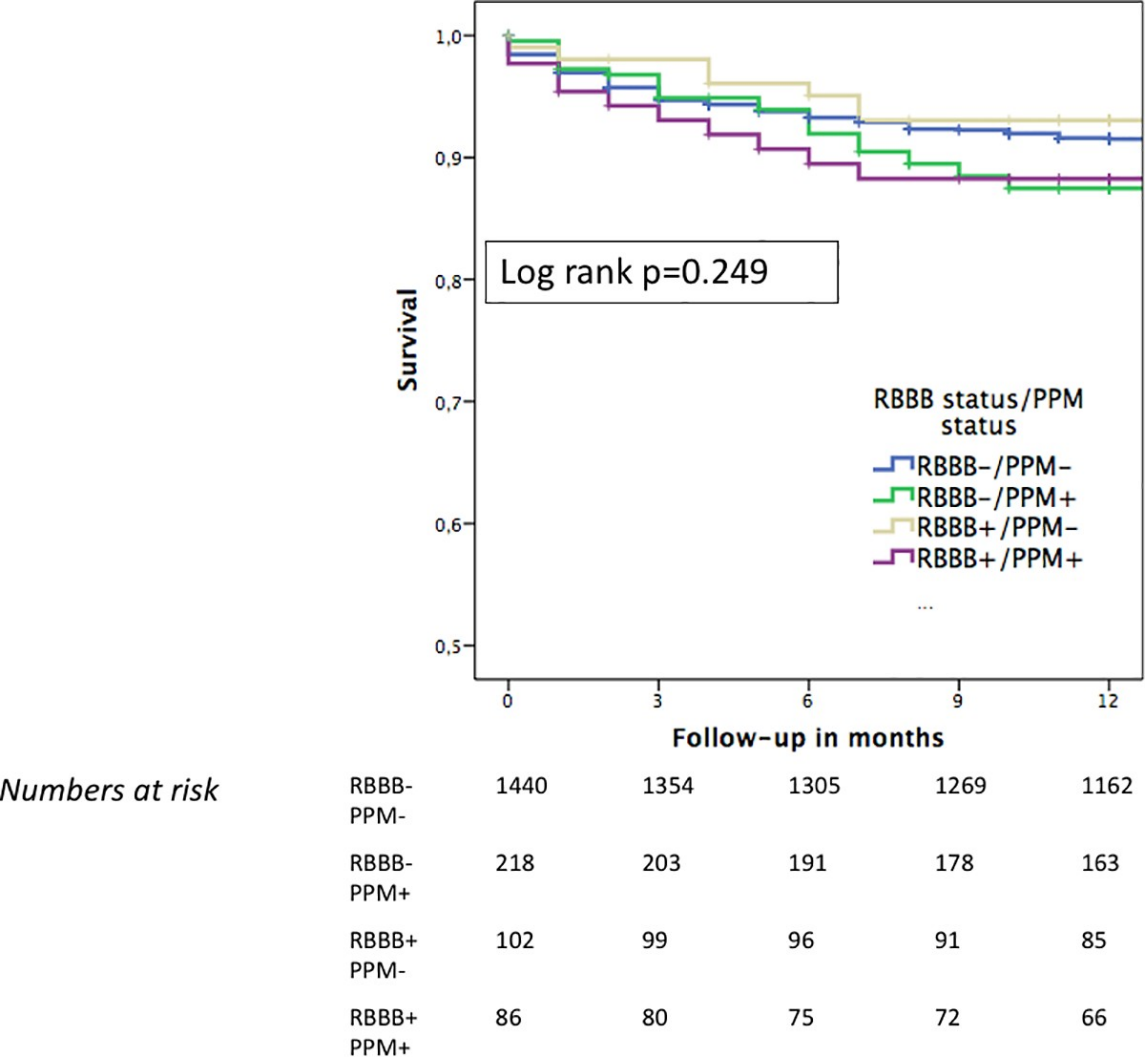
One-year cardiovascular survival in RBBB- and non- RBBB patients according to PPM implantation. During a follow-up time of 12 months, no difference in cardiovascular survival was observed between the subgroups of RBBB and non-RBBB patients with either a new PPM or no new PPM. Abbreviations: RBBB = right bundle branch block; PPM = permanent pacemaker.

At 12 months, cardiovascular death occurred in 9% of cases (166/1,846; 65.9% of all deaths) in the overall cohort and was similar for the RBBB and non- RBBB groups (9% vs. 9%) (Table 2). Further stratification according to new PPM vs. no new PPM revealed no significant impact of PPM implantation on cardiovascular survival in the Kaplan-Meier survival analysis (Figs 3 and 4).

*Table 3* shows the predictors of all-cause 1-year mortality. In the univariate Cox regression analysis, preexisting RBBB did not predict mid-term outcome (HR 1.05; 95%CI 0.70–1.56; p = 0.817). In multivariate Cox regression analysis, BMI, EuroScore II, diabetes, baseline mean transvalvular pressure, PVL ≥moderate, AKI >stage 2, new PPM, and disabling stroke after TAVI remained independent predictors of 1-year all-cause mortality.

**Table 3:** Univariate and multivariable predictors of 1-year all-cause mortality.

	Univariate HR [95% CI]	p-value	Multivariable HR [95% CI]	p-value
***Baseline characteristics***				
Age, years	1.01 (0.99–1.04)	0.237	-	-
Sex, male	1.19 (0.93–1.52)	0.178	-	-
Body mass index, kg/m^2^	0.96 (0.93–0.99)	*0*.*002*	0.94 (0.91–0.97)	*<0*.*001*
Logistic EuroScore I, %	1.02 (1.01–1.03)	*<0*.*001*	1.0 (0.99–1.01)	0.771
EuroScore II, %	1.06 (1.05–1.07)	*<0*.*001*	1.04 (1.01–1.06)	*0*.*006*
NYHA class 3 or 4	2.19 (1.46–3.28)	*<0*.*001*	1.66 (1.08–2.54)	*0*.*021*
Cardiac decompensation	2.05 (1.60–2.63)	*<0*.*001*	1.35 (1.02–1.80)	*0*.*038*
Syncope	0.99 (0.70–1.40)	0.965	-	-
Diabetes mellitus	1.55 (1.21–1.99)	0.001	1.39 (1.04–1.85)	*0*.*025*
COPD	1.29 (0.97–1.72)	*0*.*082*	-	-
Coronary artery disease	1.01 (0.79–1.30)	0.926	-	-
Prior myocardial infarction	0.84 (0.56–1.27)	0.411	-	-
Peripheral artery disease	1.60 (1.15–2.23)	*0*.*005*	1.44 (0.99–2.11)	0.06
CABG	1.17 (0.81–1.69)	0.41	-	-
Atrial fibrillation	1.77 (1.38–2.27)	*<0*.*001*	1.37 (1.03–1.84)	*0*.*034*
AV block I°	0.74 (0.53–1.06)	*0*.*097*	-	-
RBBB	1.05 (0.70–1.56)	0.817	-	-
LBBB	1.20 (0.83–1.75)	*0*.*335*	-	-
Ejection fraction, %	0.98 (0.97–0.99)	*<0*.*001*	1.01 (0.99–1.02)	0.107
Pmean, mmHg	0.98 (0.97–0.99)	*<0*.*001*	0.99 (0.98–1.0)	*0*.*006*
***Procedural/post-procedural characteristics***				
Major/life-threatening bleeding	1.88 (1.70–3.60)	*<0*.*001*	1.86 (1.07–3.23)	*0*.*027*
Major vascular complications	1.88 (1.30–2.71)	*0*.*001*	1.12 (0.67–1.87)	0.674
Disabling stroke	4.34 (2.72–6.93)	*<0*.*001*	3.98 (2.35–6.75)	*<0*.*001*
PVL ≥moderate	2.37 (1.43–3.93)	*0*.*001*	1.79 (1.00–3.20)	*0*.*049*
New PPM	1.58 (1.18–2.12)	*0*.*002*	1.57 (1.15–2.14)	*0*.*004*
AKI stage 2 or 3	3.90 (2.47–6.16)	*<0*.*001*	2.49 (1.46–4.24)	*0*.*001*

Abbreviations: CI = confidence interval; HR = hazard ratio; COPD = chronic obstructive pulmonary disease, CABG = coronary artery bypass graft, SAVR = surgical aortic valve replacement, AV block I° = atrioventricular block I°; RBBB = right bundle branch block, LBBB = left bundle branch block; Pmean = mean transvalvular pressure; PPM = permanent pacemaker; PVL = paravalvular leakage; AKI = acute kidney injury

## Discussion

The main finding of the present study is that preexisting RBBB before TAVI is not associated with increased short- or mid-term all-cause and cardiovascular mortality. Interestingly, postprocedural PPM did impact outcome in patients without RBBB but not in patients with RBBB at baseline.

The prognostic value of RBBB, either in a healthy population or in patients with preexisting cardiovascular disease, is highly controversial, with some studies showing an association with a higher mortality rate and others finding no difference in outcome compared with patients without RBBB [14–16]. While many studies identify preexisting RBBB as a strong predictor of PPM implantation after TAVI [5, 17–21], to date only two studies have investigated the role of preexisting RBBB on outcome after TAVI [7, 9]. Watanabe et al. identified preexisting RBBB as an independent predictor of cardiovascular short- and long-term mortality in 749 patients comprising a Japanese TAVI cohort treated exclusively with balloon-expandable valves [7]. Baseline RBBB rates of 13.6% were similar to the rate of 10.1% in our study population. However, in their study new PPM implantation among RBBB patients was lower (17.6%) than our PPM rates in this subgroup (45.8%). This extensive difference might only be partly explained by the exclusive utilization of the Edwards Sapien XT valve in their study, which is associated with low PPM rates ranging from 2.3% to 28.2% [22]. However, in our study the implantation rate of the self-expandable THV ACURATE neo and the precursor model Symetis was 40% (n = 761) compared with 37% (n = 697) of implanted Sapien XT/Sapien 3/Sapien 3 Ultra (S1 Table). ACURATE THVs are associated with very low PPM rates: in a 1,000-patient cohort implanted with ACURATE neo THVs Möllmann et al. reported a PPM rate as low as 8.3% within 30 days after TAVI. And recently, Husser et al. retrospectively compared PPM rates between the Sapien 3 and ACURATE neo and found a significantly lower rate in ACURATE neo patients than in Sapien 3 patients (23.1% vs. 44.6%; p = 0.016) [23]. Nevertheless, the proportion of self-expanding valves that are associated with high PPM rates, e.g. CoreValve, CoreValve Evolut, Lotus, and Portico [5, 8, 21, 24], was 23% in our study and was likely one contributing factor for the higher PPM rate. Another aspect that can be taken into account regarding the higher PPM rates in our analysis is the long study period that began in 2010 and ended in 2019. In the earlier years of TAVI, a deeper implantation technique and over-sizing of the THV in relation to the annulus were more common, both contributing factors for higher PPM rates [25, 26]. In addition, in our study there was a markedly higher rate of first-degree AV block in the RBBB cohort compared with the non-RBBB group (28.4% vs. 16.8%; p<0.001). As first-degree AV block is also a well-known predictor of PPM in TAVI patients [26], this might also have contributed to the higher PPM rate in this subgroup.

Another important aspect of the analysis of Watanabe et al. [7] is that in RBBB patients without new PPM, cardiovascular death rates were highest early after discharge, indicating that sudden cardiac death due to complete heart block might have been the leading cause of death. Indeed, development of LBBB occurs in 4–65% of TAVI procedures, depending on the valve type and implantation features like implantation depth, with mechanical insult to the conduction system as the main pathophysiological cause [18, 26, 27]. Naturally, new-onset LBBB leads to complete AVB in RBBB patients and hence to the need for PPM. The lower new PPM rate during the index hospital stay in the study by Watanabe et al. is consistent with this assumption, given the observation that late-onset LBBB occurs in up to 2.9% of cases [10, 24]. Early hospital discharge might contribute to the worse cardiovascular outcome, especially in RBBB patients without new PPM. Unfortunately, data on hospital length of stay was not reported in that study, precluding a comparison with our study or further conclusions. At mid-term follow-up, the study investigators found the highest all-cause mortality in the subgroup of RBBB patients with new PPM [7], leading to the assumption that PPM-associated factors like right ventricular pacing might be contributing factors [28]. However, long-term death rates for non-RBBB patients with new PPM were evidently lower than those of the RBBB patients, so that other factors beyond PPM-associated components could have played a role. The authors state that RBBB itself might reflect a concomitant condition associated with increased risk, although there were no differences in baseline characteristics between RBBB and non-RBBB patients in their study cohort. Indeed, a recently published meta-analysis found a higher all-cause mortality rate in patients with RBBB in the general population as well as in patients with heart disease [29]. Two older studies, however, did not find an association of RBBB with worse outcome [30, 31]. Our results contradict the conclusions of Watanabe et al. [7], since we did not observe higher death rates for RBBB patients in short- or in mid-term follow-up. One possible explanation might be that the RBBB cohort in our study had a significantly lower clinical risk score than the non-RBBB group as indicated by the EuroScore II. Presumably, a potential adverse effect of RBBB on outcome might be compensated by “healthier” patients as reflected by the risk score. Furthermore, atrial fibrillation turned out to be an independent predictor of all-cause mortality in our analysis, and this condition was significantly less frequent in RBBB patients than in non-RBBB-patients. In the analysis by Watanabe et al. logistic EuroScore I and EuroScore II were not different between the groups [7]. However, it is worth mentioning that in the Cox regression analysis for the entire cohort (*Table 3*) new PPM was identified as an independent predictor of 1-year all-cause mortality, and, as demonstrated in the Kaplan-Meier curves stratified by RBBB and PPM status, both RBBB and non-RBBB patients showed worse survival compared with RBBB- and non-RBBB patients without PPM. In the further subanalysis (see *supporting information*), it was the non-RBBB subcohort with new PPM that showed significantly worse survival compared with the non-RBBB patients without new PPM, a finding that was not observed in the RBBB cohort. The impact of new PPM on mortality in TAVI patients is highly controversial in the literature [32–34]: a meta-analysis comprising 11 studies with over 7,000 patients found no association of new PPM with higher mid-term mortality [32], and another study even demonstrated a tendency for lower cardiovascular mortality in TAVI patients with new PPM after 1 year [35], a finding that could be explained by a lower rate of sudden death due to prevention of complete heart block [35]. Conversely, PPM can have an unfavorable influence on left ventricular function, as right ventricular pacing can lead to unphysiological asynchronous cardiac contraction. Chamandi et al [34] investigated the clinical impact of PPM in TAVI patients and found a higher rate of rehospitalization due to heart failure and less improvement of left ventricular function in particular in those patients with reduced ejection fraction before TAVI; however, they found no difference in all-cause mortality after 4 years compared with TAVI patients without the need for PPM [34].

In the other study investigating the role of baseline RBBB on TAVI outcome, published by Auffret et al., baseline RBBB was present in 10.3% of patients before TAVI and was identified as an independent predictor of 30-day and long-term all-cause and cardiovascular mortality, especially in those patients who had RBBB at baseline and were discharged without PPM implantation [9]. In contrast, patients with RBBB and new PPM at discharge as well as patients without RBBB, either with or without new PPM, showed similar survival rates. However, sudden cardiac death did not occur more frequently in this particular subgroup, and the authors do not provide further explanations for possible causes of the worse outcome. In our analysis of patients stratified according to the presence of RBBB and PPM implantation after TAVI, there was a significant difference in survival at the 12-month follow-up between the four subgroups, with the worst outcome in the subgroup of patients without RBBB and new PPM, although the cardiovascular mortality was not different between the subgroups. The rather small sample size in this subanalysis, however, could have contributed to the lack of statistical significance. The same might be true for the separate analysis of survival in the RBBB and non-RBBB groups (see *supporting information*), where, in contrast to the non-RBBB group, in the RBBB group a numerically but not a statistically significant worse outcome was observed for those patients receiving a new PPM. But this remains speculative.

Our study has several limitations. First, due to the long study period of over 10 years, the study cohort bears a certain amount of heterogeneity in terms of different generations of valve prostheses, patient selection (ranging from inoperable to intermediate to low surgical risk patients), and the interventionists’ learning curve. These variations notwithstanding, preexisting RBBB is not a parameter that is influenced over time. In addition, although valve design and procedural aspects have certainly improved in recent years, studies comparing older versus latest-generation devices still showed similar PPM rates without marked reductions, as shown e.g. in the SURTAVI study with CoreValve versus Evolut R (PPM rates 25.5% vs. 26.7%) [36] or for the Edwards Sapien XT vs. Edwards Sapien 3 [37]. Secondly, due to the wide range of different prostheses used in our study with their individual risk for PPM, the comparability with other studies investigating the prognostic effect of RBBB in TAVI patients is limited. A third potential limitation may be that although the decision regarding PPM implantation was based on the current guideline recommendations, there are still different approaches between TAVI centers regarding timing and adherence to guideline recommendations, making comparisons challenging and reflecting the heterogeneity concerning PPM rates among studies, as recently shown by a meta-analysis [37]. Fourth, data on patients’ medication influencing heart rate or rhythm and hence its potential influence on PPM implantation was not available. Finally, this is a single-center study of a high-volume TAVI center with a specific distribution of prostheses used. Therefore, our results might not be easily transferable to other centers.

## Supporting information

S1 Fig Mid-term survival in the non-RBBB cohort stratified by PPM status. In non-RBBB patients with new PPM after TAVI mid-term survival is worse than that of patients without new PPM. Abbreviations: RBBB = right bundle branch block; PPM = permanent pacemaker.
(TIFF)

S2 Fig Mid-term survival in the RBBB cohort stratified by PPM status. In RBBB patients PPM status had no significant impact on survival rate at one year. Abbreviations: RBBB = right bundle branch block; PPM = permanent pacemaker.
(TIFF)

S1 Table Distribution of THVs. Distribution of the different THVs in the total cohort, non-RBBB cohort, and RBBB cohort.
(DOCX)
