# Supplementary figures and images for: Right bundle branch block is not associated with worse short- and mid-term outcome after transcatheter aortic valve implantation
Source: PLoS One. 2021 Jun 16;16(6):e0253332. doi: 10.1371/journal.pone.0253332 (PMC8208572; doi:10.1371/journal.pone.0253332)

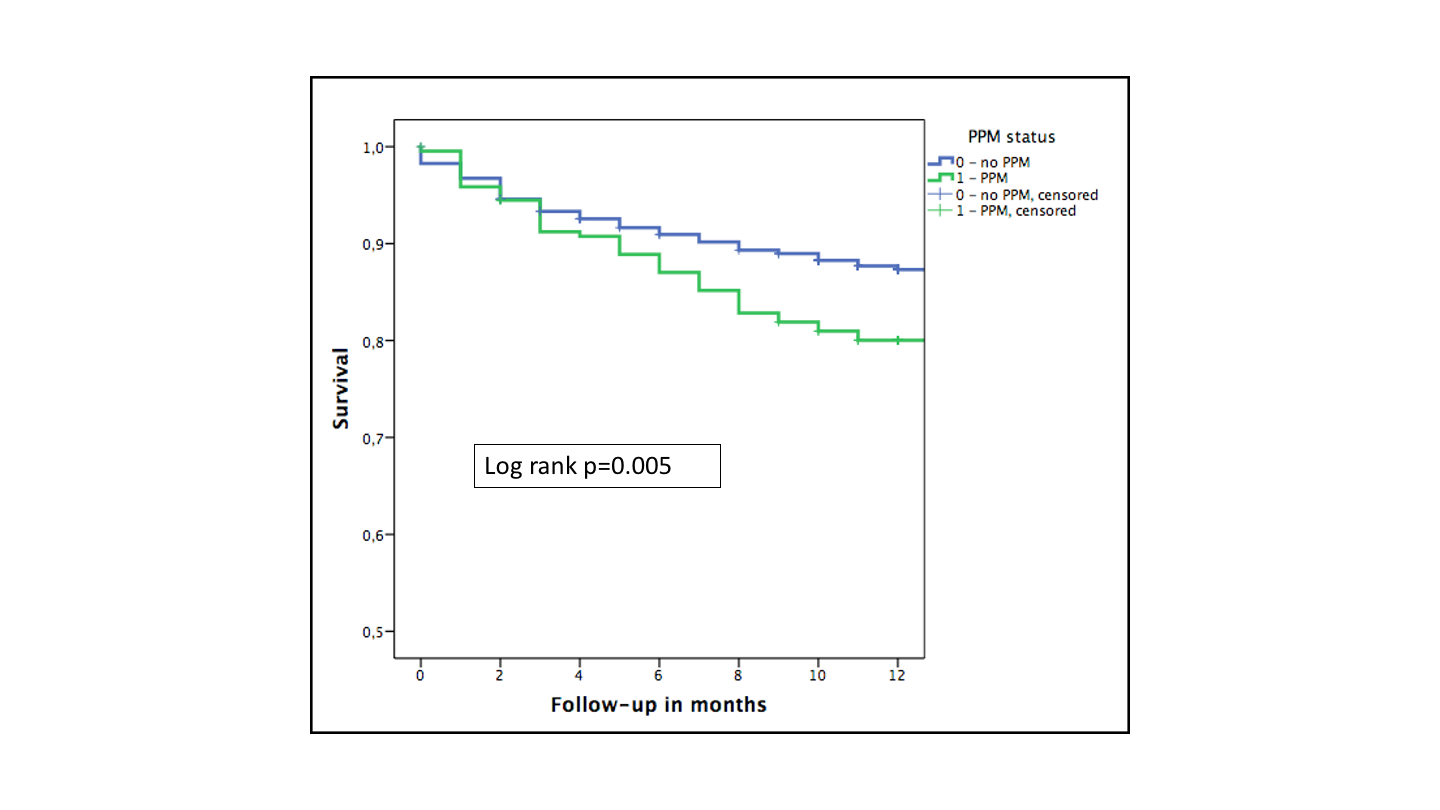

Supplement: S1 Fig — In non-RBBB patients with new PPM after TAVI mid-term survival is worse than that of patients without new PPM. Abbreviations: RBBB = right bundle branch block; PPM = permanent pacemaker. (TIFF) [file pone.0253332.s001.tiff]

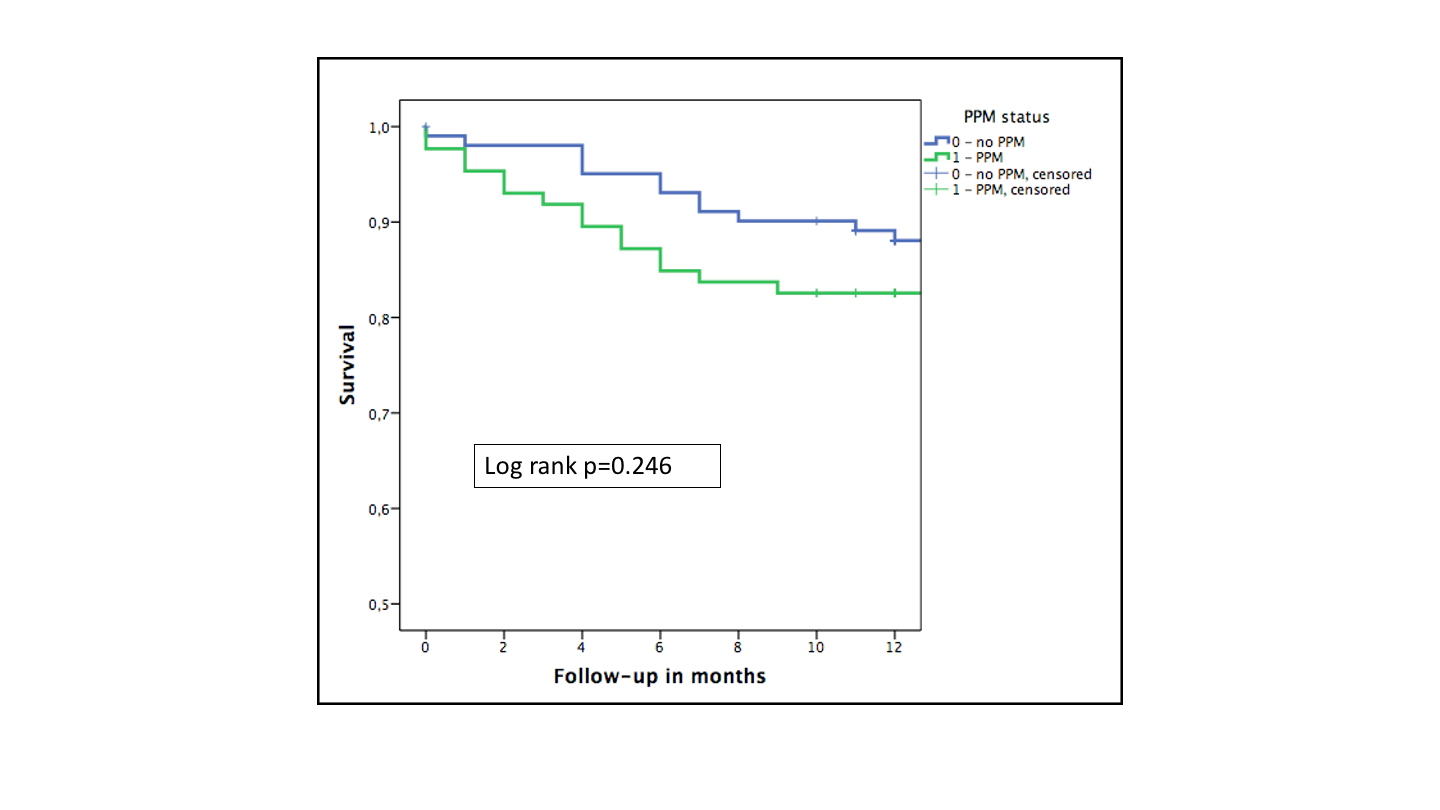

Supplement: S2 Fig — In RBBB patients PPM status had no significant impact on survival rate at one year. Abbreviations: RBBB = right bundle branch block; PPM = permanent pacemaker. (TIFF) [file pone.0253332.s002.tiff]
