# Supplementary material for: Right bundle branch block is not associated with worse short- and mid-term outcome after transcatheter aortic valve implantation
Source: PLoS One. 2021 Jun 16;16(6):e0253332. doi: 10.1371/journal.pone.0253332 (PMC8208572; doi:10.1371/journal.pone.0253332)
Supplement: S1 Table — Distribution of the different THVs in the total cohort, non-RBBB cohort, and RBBB cohort. (DOCX) [file pone.0253332.s003.docx]

**S1 Table.** Distribution of THVs

| **THV** | **Total cohort**  **(n=1,891)** | **Non-RBBB**  **(n=1,701)** | **RBBB**  **(n=190)** |
| --- | --- | --- | --- |
| *CoreValve* | 176 (9.3) | 162 (9.5) | 14 (7.4) |
| *Sapien XT* | 100 (5.3) | 91 (5.3) | 9 (4.7) |
| *Symetis ACURATE* | 43 (2.3) | 42 (2.5) | 1 (0.5) |
| *Portico* | 190 (10) | 179 (10.5) | 11 (5.8) |
| *Sapien 3* | 592 (31.3) | 520 (30.6) | 72 (37.9) |
| *Symetis Neo* | 684 (36.2) | 618 (36.3) | 66 (34.7) |
| *CoreValve Evolut* | 49 (2.6) | 39 (2.3) | 10 (5.3) |
| *Lotus* | 18 (1) | 16 (0.9) | 2 (1.1) |
| *ACURATE Neo* | 34 (1.8) | 31 (1.8) | 3 (1.6) |
| *Sapien 3 Ultra* | 5 (0.3) | 3 (0.2) | 2 (1.1) |

Distribution of the different THVs in the total cohort, non-RBBB and RBBB cohorts

Values denote number (%).

Abbreviations: THV= transcatheter heart valve; RBBB= right bundle branch block
